# Knowledge, attitudes and practices of HIV-positive patients regarding disclosure of HIV results at Betesda Clinic in Namibia

**DOI:** 10.4102/phcfm.v5i1.409

**Published:** 2013-02-12

**Authors:** Penelope Tom

**Affiliations:** 1 Faculty of Health Sciences, University of the Witwatersrand, South Africa

## Abstract

**Background:**

This study examined the practices, knowledge, attitudes, and the reasons for disclosure and non-disclosure of HIV-positive patients with regard to the disclosure of HIV results at Betesda Clinic in Windhoek, Namibia.

**Objectives:**

The objectives of the study were to determine knowledge, attitudes, and practices of HIV-positive patients regarding the disclosure of HIV status at Betesda Clinic in Namibia, and to determine the reasons for disclosure and non-disclosure.

**Methods:**

This was a cross-sectional descriptive study and 263 HIV-positive patients were enrolled in the study.

**Results:**

Analyses revealed that knowledge on disclosure was good, with 68% who thought it was important. The majority (73%) have disclosed and 60% disclosed within 1 week of receiving their results. The most common reasons for disclosure were that 32% needed help, 25% wanted his or her partner to go for testing, and 20% wanted to let relatives know. Reasons for non-disclosure were mainly the fear of gossip (79%). Seventy-three per cent had disclosed to their partners, and 23% had disclosed to more than one person. People's reactions were supportive in 43%, whereas 29% understood, 9% accepted and 6% were angry. Upon disclosure 40% received help, 24% of partners were tested, 23% received psychological support and 5% were stigmatised. Disclosure was higher amongst the married and cohabitating.

**Conclusion:**

The attitude was positive with regard to knowledge of disclosure, with most participants thinking that disclosure was important and good. The attitudes and actual practices of disclosure were encouraging; however, people are disclosing only to trusted individuals in the society and the fear of stigma is still present although the actual stigma was very low.

## Abstrait

**Prise de conscience, attitudes et pratiques des patients séropositifs concernant la divulgation des résultats de leurs tests de dépistage du VIH au centre médical de Betesda, en Namibie**

**Contexte:**

Cette étude a examiné les pratiques, la prise de conscience, les attitudes et les raisons de divulgation ou de non-divulgation des résultats des tests de dépistage du VIH de la part de patients séropositifs au centre médical de Betesda, à Windhoek, en Namibie.

**Objectifs:**

L’étude avait pour objectif de décrire la prise de conscience, les attitudes et les pratiques de patients séropositifs concernant la divulgation de leur statut sérologique au centre médical de Betesda, en Namibie, et de déterminer les raisons de leur divulgation ou non-divulgation.

**Méthodologie:**

263 patients séropositifs ont participé à cette étude descriptive transversale.

**Résultats:**

Selon les analyses, la prise de conscience du bénéfice de la divulgation était bonne, 68% des patients trouvant que c’était important. La majorité des participants (73%) a révélé son statut et 60% l'ont fait dans la semaine qui a suivi la nouvelle de leurs résultats. Les raisons pour lesquelles les patients divulguaient leur statut étaient que 32% avaient besoin d'aide, 25% voulaient que leur partenaire aille se faire tester, et 20% voulaient que les membres de leur famille soient informés. Les raisons pour lesquelles les patients ne divulguaient pas leur statut étaient principalement la crainte des commérages (79%). Soixante-treize pour cent ont révélé leur statut à leurs partenaires, et 23% l'ont révélé à plus d'une personne. Les réactions des gens ont été les suivantes: sympathie à 43%, compréhension à 29%, acceptation à 9% et colère dans 6% des cas. Après la divulgation, 40% ont reçu de l'aide, 24% des partenaires ont été testés, 23% ont reçu un soutien psychologique and 5% ont été stigmatisés. La divulgation était plus fréquente chez les patients mariés ou en concubinage.

**Conclusion:**

La prise de conscience de la divulgation a bien été assimilée, la plupart des participants trouvant que la divulgation avait été importante et bénéfique. Les attitudes et la pratique réelle de la divulgation se sont avérées encourageantes; cependant, les gens ne font leur révélation à la société qu’à des individus de confiance et la peur de la stigmatisation reste encore présente, bien qu'elle reste très faible dans la réalité.

## Introduction

### Key focus

Human immunodeficiency virus prevalence is increasing with more people being infected despite informative messages and advertising.^1^ Namibia has one of the highest HIV and AIDS prevalence rates in the world (21.3% as at the end of 2003). Two hundred and ten thousand (210 000) people were estimated to be living with HIV or AIDS, that is, more than one in five adults. In 2003 an estimated 16 000 Namibians died of HIV-related diseases, the leading cause of death amongst adults and children.^2^ Is this because HIV is stigmatised or is people's knowledge about HIV inadequate? Are people disclosing and is there a change in attitude towards the virus? These were some of the questions which needed answers. In Namibia where tradition is highly respected, culture is diverse and HIV is increasing, no published studies on disclosure were found and yet disclosure is considered a key element in the treatment and prevention of HIV.

Another question was whether individuals were at peace after disclosure. The researcher had a patient who had been on antiretroviral treatment for 5 years. In 2008 he was involved in a dispute and suffered severe chest injuries. On admission he could not ask his wife to bring his antiretroviral medication as he had not disclosed his status to other people. He thus defaulted on his treatment for 1 month and the viral load became detectable after being undetectable. This may have contributed to non-healing wounds 6 months later, and his was probably not an isolated case.

Prevention of the spread of HIV depends on widespread testing to detect infection and management or treatment of HIV-positive individuals. Disclosure has a number of benefits for the individual which includes opportunities for social support, improved access to medical care, increased opportunities to discuss and implement risk reduction with partners, opportunities to take leadership roles in the community, and increased opportunities to plan for the future.^3^ A considerable amount of literature documents the experience of stigma and discrimination but fails to document the benefits of disclosure.^4^

Disclosure is a difficult and challenging process. One of the biggest concerns is confidentiality. Can the confidant(e) be trusted to keep the information secret? No literature was found on people's knowledge regarding the importance of disclosure, but people are aware that disclosure can have positive as well as negative effects. People living with HIV find it daunting to disclose their status for a number of reasons; it will no longer be a secret, and there is a fear of rejection and break-up of relationships. Other reasons are loss of economic support, blame, abandonment, physical and emotional abuse, discrimination and disruption of family relationships.^5^

These fears are a natural part of the risk because stigmatisation may and does occur.^6^ Stigma can be in the form of internalised stigma which is the acceptance of the lived situations, and the self-discrimination that a person endures over time, or externalised where other people stigmatise an individual. Stigma can be experienced at individual, family, community and societal level. Individuals avoid being tested, which in turn prevents people from recognising that they or their family members are HIV-positive and people will thus not seek care, support and treatment. Fear of stigma may also cause people to mislead others, and impede people from using barrier methods of protection, thereby increasing transmission. It may cause people to refrain from accessing quality care, it can hinder people from gaining access to benefits, and it can increase morbidity and mortality with increased social insecurity.^7^

People stigmatise others because they are insecure, afraid and ignorant, and because they lack knowledge.^8^ Society needs continuous education for the cycle of stigma and discrimination to end. When HIV and AIDS were first detected, it was closely associated with certain groups within society which were taboo and so the HIV-positive were ostracised and branded. Those who stigmatised may have thought that by doing so they could keep HIV away from themselves and that they would be safe from HIV. It is no longer necessary to be victims and HIV-positive persons should be encouraged to learn to let go of internal stigma, to accept themselves and be confident, to disclose and to believe that they deserve love and support from the community.

### Significance of the study

This study focused on HIV-positive patients so that the actual practices and attitudes of the society as experienced by the patients were measured. Other similar studies focused on specific groups, for example patients with tuberculosis, pregnant women, men who have sex with men, and drug users.^9, 10^

## Ethical considerations

Permission to conduct the study was obtained from Betesda Clinic authorities; ethical clearance was obtained from the Post Graduate Committee and Human Research Ethics Committee of the University of the Witwatersrand. Participation was voluntary, an informed consent was signed and refusal to participate did not affect the patients’ treatment at the clinic. Confidentiality and privacy were maintained by providing a private room for completing the questionnaire.

## Methods

### Study design and participation

The data was collected from 01 March 2010 to 15 April 2010 as part of a cross-sectional study at Betesda Clinic in Windhoek, Namibia. Betesda Clinic is a private clinic and provides primary health services to the community, mainly to those with medical insurance. The clinic has 1013 known HIV-positive patients and those who were 18 years or older were eligible for the study. All consenting HIV-positive patients who visited the clinic during the study period were entered into the study. Consecutive patients who fulfilled the inclusion criteria were added to the study group until the required size was obtained. All patients, no matter what ails them, wait in the same queue and there is no separate HIV clinic. Given 1013 patients, a sampling error of 5%, the ability to detect a difference of 50% and a 95% confidence level, the required sample size was 245. A 10% inflation fraction was added giving a sample size of 269. Six respondents were lost because they were too sick or in a hurry, which resulted in a sample size of 263. A questionnaire was used to collect data. The data were analysed by using the Epi Info Statistical Software package. An expert statistician assisted with the analysis of the data. Univariant analysis was performed to calculate frequency proportions and means.

## Results

The study population comprised 263 respondents of varied marital status (Table 1). Fifty-five per cent were in the age group 38–47 years, 21% were 48–57 years, 16% were 28–37 years, 6% were 58–67 years, 3% were 18–27 years and none were older than 67 years. With regard to the educational level, 49% were from Grade 8 to 12, 38% were from Grade 1 to Grade 7, 7% had higher education which comprised college and university level and 6% never attended school.

**TABLE 1:** Marital status composition of the study group.

Marital status	%	*f*
Married	41.5	109
Single	28.1	74
Cohabitating	27.0	71
Widow or widower	2.3	6
Divorced	1.1	3

**Total**	**100**	**263**

*f*, Frequency.

**TABLE 2:** What do you think about disclosure?

Thoughts on disclosure	%	*f*
Good	41	107
Important	14	36
Not good	13	35
Okay	11	29
Difficult	5	13
Depends	3	8
Helps	2	6
Do not like it	2	5
Not sure	2	4
Problem	2	5
Others (do not know, scary, nothing, not important, necessary, stigma)	5	15

**Total**	**100**	**263**

*f*, Frequency.

The question, ‘What do you think about disclosing HIV results?’ was asked. Sixty-eight per cent had positive thoughts about disclosing their status, 27% had negative thoughts and 5% were neutral.

**FIGURE 1:**
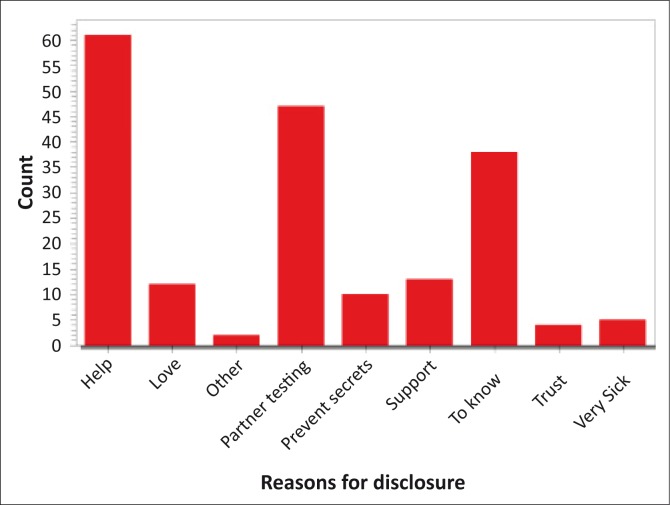
Reasons for disclosure.

The next question was, ‘What were your reasons for disclosing?’ and the response was noted (Figure 1). Reasons for disclosing were that they needed help (32%; *n* = 61), 25% (*n* = 47) wanted their partner to go for testing, 20% (*n* = 38) wished to let relatives know why they were sick, 7% (*n* = 13) wanted psychological support and 6% (*n* = 12) wanted to disclose because they loved the people to whom they were disclosing.

‘What were your reasons for not disclosing?’ was asked to all the participants and the response was noted (Table 3). Reasons for not disclosing were fear of the community gossiping (the talking and the finger pointing, 79%), and 3% said it was their secret.

**TABLE 3:** Reasons for not disclosing.

Reasons for not disclosing	Not disclosed		Disclosed	
	
	%	*n*	%	*n*
Fear of the community gossiping	20	54	59	155
Fear divorce	1	3	-	-
My secret	3	9	-	-
No trust	1	3	3	8
Relatives will worry	0.4	1	-	-

**Total**	**25.4**	**70**	**62**	**163**

*n*, Given as number of patience.

The response to, ‘If you have disclosed your status to anyone, to who have you disclosed?’ was tabulated (Table 4). Seventy-three per cent of the respondents had disclosed their results. Those who had disclosed, had disclosed to the following people: 73% had disclosed to their partners whilst 23% had disclosed to more than one person, 15% had disclosed to their brothers, 21% had disclosed to their sisters, 8% had disclosed to their mothers, 4% had disclosed to a friend, 4% had disclosed to their children, 4% had disclosed to others (uncle, employer, grandfather and pastor).

**TABLE 4:** To whom have you disclosed?

To whom have you disclosed?	%	*f*
Spouse or Partner	73	141
Family (brother, mother, sister, child, uncle, niece, grandfather)	40	76
Friend	4	8
Employer	0.5	1
Pastor	0.5	1

**Total**	**-**	**227**

*f*, Frequency.

### In what time span did you disclose?

The time span of disclosure was, 60% within 1 week of knowing their results, 11% within 3 months, 14% from 3 to 6 months, 12% from 6 to 12 months, and 3% after 12 months.

**FIGURE 2:**
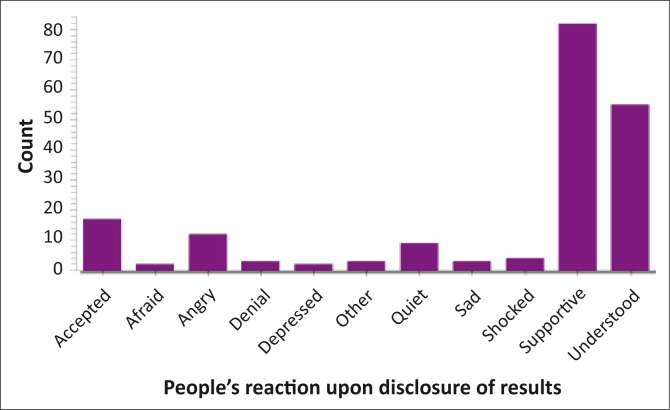
How did people you disclosed to react to your results?

‘How did people you disclosed to, react to your results?’ was asked and the response was illustrated (Figure 2). The reactions of people disclosed to were as follows: 43% (*n* = 82) were supportive, 29% (*n* = 55) understood, 9% (*n* = 17) accepted, 6% (*n* = 12) were angry, 5% (*n* = 9) were quiet. Of those who had disclosed, 96% (*n* = 184) did not regret disclosing their status.

**FIGURE 3:**
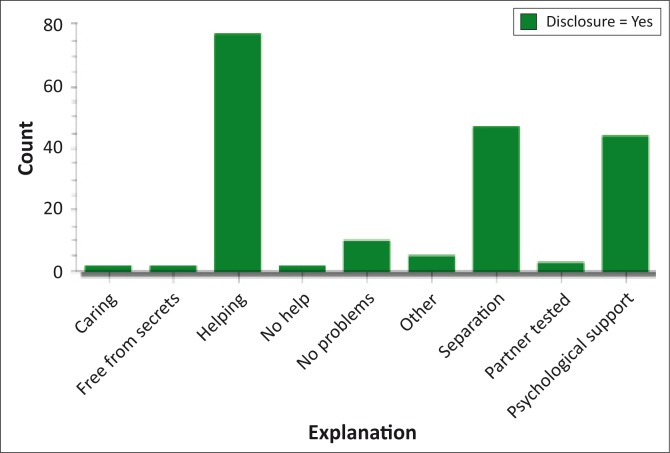
Explain why you regret or do not regret disclosing your results.

To the question, ‘Explain why you regret or do not regret disclosing your results?’ the response was as follows: of those who had disclosed, 96% (*n* = 184) did not regret disclosing their results, 3.5% (7) regretted disclosure and 0.5% (1) were not sure. Those who were treated positively had the following responses from the people they disclosed to: 40% (*n* = 77) provided help, 24% (*n* = 47) of the respondents’ partners’ were tested, 23% (*n* = 44) were given psychological support, 5% (*n* = 10) encountered no problems. Those who received negative treatment received it in the form of separation from relationships (2% [*n* = 3]), and 3% (*n* = 6) comprised other forms which included fighting, gossiping, and no help.

**FIGURE 4:**
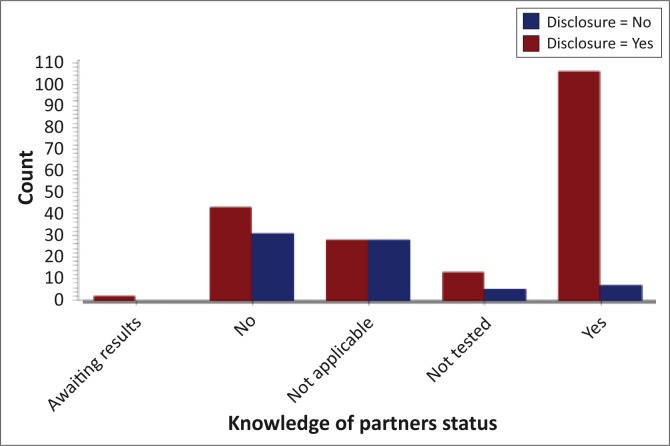
Knowledge of Partners’ Status and Disclosure.

Of all the participants, 43% (*n* = 113) knew their partner's status, and of these 94% (*n* = 106) had disclosed. Twenty-eight per cent (*n* = 74) did not know their partner's status, and of these 58% (*n* = 43) had disclosed. Seven per cent (*n* = 18) of the partners were not tested yet, and of these 72% (*n* = 13) of the respondents had disclosed (Figure 4).

**FIGURE 5:**
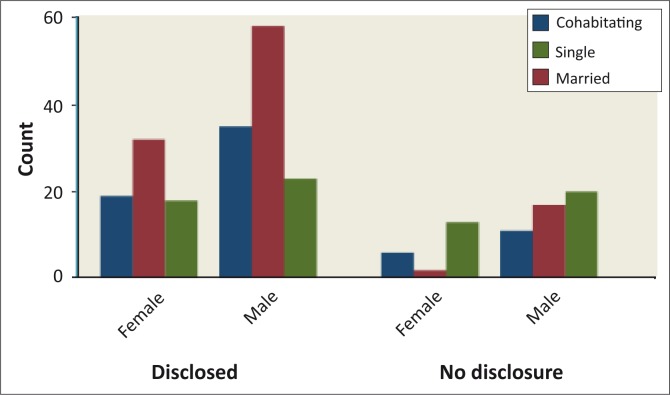
Patients-disclosure by Marital Status and Gender.

Of the married participants, 83% (*n* = 90) had disclosed, whilst 17% (*n* = 19) had not; 76% (*n* = 54) of the cohabitating participants had disclosed, whilst 24% (*n* = 17) had not, and 55% (*n* = 41) of participants with single status had disclosed whilst 45% (*n* = 33) had not disclosed. Non-disclosure was found to be higher amongst single participants and divorced participants than other groups, whereas disclosure was highest amongst the married and cohabitating participants (Figure 5).

## Discussion

Disclosure is considered an essential part of managing HIV. Social support provided by family and friends has both practical and emotional components, helping to build new positive identities for the positive individuals with greater acceptance of their status and to provide a buttress against insults from neighbours. Analysis has shown that positive living is closely associated with a better health outcome.^11^

Of the respondents, 73% had disclosed which implies that stigma is reduced and people are slowly opening up. Similar results were found in a study carried out in Tshwane, South Africa, where stigma was found to be significantly lower than what was perceived to be present in the community.^12^ Respondents were aware that without disclosure there was neither help nor support. Individuals will choose to share information if the rewards are greater than the costs of disclosing. More than half (60%) of the respondents disclosed their status within 1 week of testing. This was very surprising, because in most of these families the husbands stay in town to work whilst the wives and children stay in the rural areas where they grow their own food and they only meet during holidays.^13^ This means that most of those infected were trying to pass on these results to their families as early as possible, which is encouraging.

In this study, thoughts on disclosure were positive. When respondents were asked what they thought about disclosure, the majority (68%) had positive thoughts. Positive respondents said it was good, important, it helps and it is necessary. The majority understood the concept of disclosure. Less than a third (27%) had negative thoughts. These thoughts were expressed as being not good, not important, causing problems, scary and stigmatising, whilst 5% were not sure. These few individuals would benefit from education about the importance of disclosure. This can be performed during counselling sessions.

All the respondents did not disclose to everyone in the community, but only to trusted selected individuals. Seventy-three per cent disclosed to their partners, 21% disclosed to their sisters and 23% disclosed to more than one person. Other people they disclosed to were a mother, brother, children, uncle, grandfather, pastor, friends and employer. The majority of the respondents disclosed for a reason; almost 100% had a reason to disclose whilst 0.5% disclosed without a reason. The majority of the respondents disclosed because they wanted help. Other reasons for disclosure were to encourage their partner to go for testing, they were angry, they wanted their relatives to know, they were very sick, they wanted to prevent secrets, and for the experience of sharing and love. Reasons for non-disclosure were fear of people talking, no trust, they did not want relatives to worry about them, fear of divorce and private issues. In a study conducted in Southwest Ethiopia, perceived positive outcome expectations were most frequently associated with disclosure.^14^

Similar findings were seen in a study in South Africa; patients’ decision to disclose was based on expectations of support from family and friends, personal preparedness and trust, whilst fear of being labelled restricted disclosure.^15^ This was observed in China from illness narratives; fear of isolation and the urge to protect close family members hindered disclosure.^16^

Of those who disclosed, 81% received a positive reaction from the people to whom they had disclosed, with 43% receiving a supportive reaction, and 29% of the people to whom they disclosed were understanding. Other reactions were acceptance, prayer, help, sympathy, worry, denial, fright, anger and shock. Upon asked whether the respondents regretted disclosure, 96% had no regrets. All of them were supported by the people they had disclosed to; 40% were given help, 25% of the partners went for testing, 23% received psychological support and 1% were supported with prayers. Only a few (4%) regretted disclosing because they had received negative treatment from the people they disclosed to; this was in the form of domestic violence, separation from relationships and gossiping.

Ignorance is fading and people are beginning to understand disclosure. Those who did not disclose said they were afraid of people talking and finger pointing. Fear of stigma was still high although stigma experience was minimal. In a review article similar findings were recorded, 54% – 94% feared abandonment, discrimination and violence, but only 4% – 15% reported violence.^17^ In a project carried out in Katutura, Namibia, women testing negative were found to have higher levels of disclosure of their results to their partners than those testing positive, the latter fearing reactions. Those who had disclosed, did so to close family members and relatively soon after being told. When HIV-positive women disclosed, contrary to their fear of blame, they reported receiving support from their families.^18^

Eighty-three per cent of the married and 76% of the cohabitating had disclosed to their partners or spouses. If one's partner discloses, one is more likely to be tested and to disclose. This is very important in relationships because it becomes easier to negotiate safer sex, to discuss future pregnancies, and compliance improves because there is no need to hide medication or to fabricate a lie. In a study carried out in Cape Town a close association between having not disclosed HIV status to sex partners and engaging in behaviour associated with a high risk of HIV transmission, was observed. People who had not disclosed reported more sex partners and more unprotected vaginal and anal intercourse.^19^ Of those who knew their partner's status, 94% had disclosed. A few of the respondents were still keeping their results secret although they knew their partner's status. Identifying these few individuals at clinics every time they come for follow-up is important. Lack of awareness of a partner's serostatus may result in transmission of HIV, especially within serodiscordant couples because protective behaviours may not be adopted.^20^

Non-disclosure was found to be higher amongst single and divorced individuals, whilst disclosure was highest amongst the married and cohabitating respondents. When people are single or divorced, the relationships are insecure and disclosing HIV-positive results can be risky because the response may be unpredictable. In a study carried out in South-West Ethiopia, Deribe found that individuals who were living in the same house with their partners were 9.2 times more likely to disclose their HIV-positive results compared to those who did not live in the same house.^21^

When respondents were asked whether they would want their family members to keep their results a secret, 60% of those who had disclosed said they would not want their family members to keep their results a secret. This is understandable; if one discloses, one would expect the person one disclosed to, to do the same. Surprisingly, 32% of those who had disclosed said they would want their family members to keep their results a secret. Some people believe in respecting other people's wishes. Of those who did not disclose, 24% said they would not want their family members to keep their results a secret. Without disclosure there can be no support from the family or community.

There was not much difference in disclosure amongst the age groups. Non-disclosure ranged from 24% to 37% across the age groups. Compared to other age groups, the 18–27 age group was small (3%); either this young age group was not coming to be tested or they were going somewhere else or they were taking precautions so as not to be infected. This is a critical age group, where relationships and families are started and babies are born, and it is crucial that this age group is tested. They were going probably where free testing and treatment was given. HIV-testing uptake amongst young age groups was found to be low in a study conducted in Nairobi, Kenya.^22^ Educating young people, thereby increasing awareness and the importance of HIV status will help to prevent the spread of HIV.

Counselling should be an ongoing process and should be available to everyone whether HIV-positive or not. Disclosure should be encouraged as a chronically ill person without physical, financial and emotional support from friends, relatives and the community, will find it difficult to manage on his (or her) own. Internal stigma, shame or fear of discrimination can lead to HIV-positive people living an isolated life, with a shrinking social circle. Further consequences are the avoidance of HIV-related topics, uneasiness when HIV-related issues are being discussed and a feeling of obligation amongst mothers to breastfeed, thereby exposing their babies to HIV-infection because alternative methods of feeding may create suspicion in the family or community.^23^

In resource-limited settings like Namibia, free drama documentaries such as the South African movie ‘Yesterday’, can be shown in the waiting rooms in clinics. Dramas with an African cast and in context can have an impact in countries like Namibia. The formation of support groups like ‘HIV-positive anonymous’ where HIV-positive people meet, share their experiences, support each other and develop friendships, should be initiated in clinics and joining such groups can form part of counselling.

### Limitations of the study

The results may not extrapolate to the public sector, because the study was conducted in a private clinic where patients on either medical insurance or those who could afford to pay, were seen, which was a limitation. Another problem in retrospective recollection studies is recall bias; respondents may have problems remembering, especially if the information is collected a long time after the incident.

### Recommendations

Counselling should be offered by the doctors, nurses or counsellors as an ongoing process in clinics every time HIV-positive patients come for a review or follow-up. Posters and pamphlets should be available in clinics promoting disclosure, discouraging stigma, and highlighting the effects of stigmatisation. HIV-anonymous group formation can be initiated in clinics.
